# Dynamics in the Transimpedance Matrix and Electrically Evoked Compound Action Potentials in Cochlear Implant Users With a Lateral-Wall Electrode Array

**DOI:** 10.1097/AUD.0000000000001729

**Published:** 2025-09-10

**Authors:** Topi Jutila, Saku T. Sinkkonen, Samuel Söderqvist, Ville Sivonen

**Affiliations:** 1 Department of Otorhinolaryngology–Head and Neck Surgery, University of Helsinki and Helsinki University Hospital, Helsinki, Finland; 2 Department of Otorhinolaryngology–Head and Neck Surgery, University of Turku and Turku University Hospital, Turku, Finland.

**Keywords:** Cochlear implant, Electrically evoked compound action potential, Electrode impedance, Intracochlear electric field, Neural response telemetry, Transimpedance matrix

## Abstract

**Objectives:**

In patients with cochlear implants, tools for measuring intracochlear electric environment as well as neural responses to electrical stimulation are widely available. This study aimed to investigate the possible correlation of changes in the responsiveness of the auditory nerve measured by neural response telemetry with changes in the peak and spread of the intracochlear electric field measured by transimpedance matrix (TIM) in patients implanted with straight electrode arrays.

**Design:**

In this retrospective study, we analyzed a cohort of 144 ears of 113 consecutive patients who were implanted with Slim Straight electrode array (Cochlear Ltd.) between January 2019 and May 2022. Thirty-four ears of 30 patients had both intra- and postoperative data available for TIM and neural response telemetry thresholds (T-NRT). The postoperative measurements for TIM and T-NRT took place on average 101 (median: 42; range: 22 to 709) and 126 (median: 62; range: 23 to 427) days postoperatively, respectively. In addition, clinical electrode impedances were measured intraoperatively at two time points in 123 ears and postoperatively during all programming visits.

**Results:**

Electrode impedances gradually increased over time after the onset of the cochlear implant use, and this was more pronounced in the basal part of the electrode array (*p* < 0.001). Postoperative T-NRTs decreased significantly from the intraoperative measurement (*p* < 0.001). Postoperative T-NRT between different time points increased slightly in the apical section (*p* = 0.03) but remained stable in the middle and basal sections of the electrode array. Postoperative intracochlear electric field spread narrowed significantly in the middle section (*p* = 0.001), and electric field peak value increased significantly in the basal section (*p* = 0.003) compared to intraoperative values. The increased electric field peak value and the decreased T-NRT between intra- and postoperative measures in the basal section of the electrode array had a significant negative correlation (*ρ =* −0.42, *p* = 0.02).

**Conclusions:**

A novel finding of this study was that the increased postoperative peakedness of the intracochlear electric field was correlated with the increased responsiveness of the auditory nerve in the basal section of the electrode array. The narrower and more peaked intracochlear electric field may be due to fibrous tissue formation around the electrode array. Aligned with the onset of electrical stimulation and the general time course of intracochlear fibrous tissue formation after cochlear implant surgery, a long-term increase in electrode impedances was found, which was more pronounced in the basal section of the array.

## INTRODUCTION

Multichannel cochlear implants (CIs) are widely used to restore hearing in patients with severe-to-profound hearing loss in both children and adults. CI rehabilitation includes preoperative multidisciplinary evaluation and planning, surgical procedure, device activation typically within a few weeks after the surgery, and follow-up visits. During follow-up visits, the professional programs the CI, that is, adjusts the electric current to directly stimulate the spiral ganglion cells along the CI electrode array, as well as counsels the patient. After the insertion of the electrode array into the cochlea during CI surgery, it is customary to measure the device function and the electrical environment within the cochlea, as well as neural responses elicited with the device to verify that the auditory nerve (AN) responds to electrical stimulation.

Overall electrode impedances that are clinically available in CIs reflect the impedance to the flow of electric current from the stimulated intracochlear electrode contact to a return electrode, which, depending on the measurement mode, can be one or two extracochlear electrodes, or alternatively, one or all other intracochlear electrodes. Clinical impedances are affected by the resistivity of the electrode contacts and the lead wire, the electrode-tissue interface as well as the resistivity of the adjacent fluid and surrounding tissue (Tykocinski et al. 2005; Goehring et al. 2013). Measuring impedances intraoperatively after electrode insertion helps ensure the integrity of the electrode contacts. During the programming visits, impedances guide the CI programming in terms of compliance limits, and they affect the power consumption of the implant.

Long-pulse clinical impedances can be decomposed into access resistance and polarization impedance. Access resistance represents the resistivity of the bulk tissue surrounding the electrode array, and it is likely associated with the development of fibrous tissue around the electrode (Tykocinski et al. 2005; Newbold et al. 2010). Access resistance can be further split into a near- and far-field component, the former representing a rise in electric potential in a small region in the vicinity of the stimulating electrode contact, and the latter representing resistance outside this small region and along the electrode array. As a biomarker, the magnitude of the near-field component is positively correlated with the amount of device use (i.e., electrical stimulation) across the electrode array. In addition, longitudinal increases in the far-field component are more pronounced in the basal part of the cochlea, where fibrous tissue formation is also greater (Leblans et al. 2022). Polarization impedance, on the other hand, represents the complex capacitive behavior of the electrode-electrolyte interface, and it is affected by a layer of proteins on the surface of the electrode as the surfaces of biomaterials adsorb a protein layer within seconds to minutes after tissue contact (Tang & Eaton 1999). Electric current, however, dispels this protein layer, hence decreasing the polarization impedance (Tykocinski et al. 2005). In a clinical setting, the effect of feeding a relatively high electric current to the electrode-tissue interface is called conditioning.

By determining the electrically evoked compound action potential (eCAP), the response of the AN to electrical stimulation can be measured individually for each contact of the CI electrode array both intra- and postoperatively. eCAPs have an important role to ensure that the electric field (EF) generated by the CI results in an appropriate neural response in the AN. Indeed, present eCAPs along the electrode array during surgery after the electrode is inserted into the cochlea help checking both CI and AN integrity. With Cochlear Ltd. (Sydney, NSW, Australia), neural response telemetry (NRT) is the protocol for measuring eCAPs, which most often are assessed as NRT thresholds (T-NRT), that is, the minimum current to evoke an action potential. Because of the introduction of the Nucleus Freedom device, an automated NRT (AutoNRT) enabled automated interpretation of the eCAPs. Due to AutoNRT, eCAPs have become more widely utilized in CI programming. The eCAP threshold profile along the electrode array facilitates CI programming, especially for young children or other patients who may provide inconsistent behavioral responses in setting their stimulation levels, because the T-NRTs and the CI stimulation profiles are correlated (Brown et al. 2000; Lai et al. 2009). Besides programming, measuring eCAPs postoperatively can be used to monitor the recipient (van Dijk et al. 2007). In addition, AutoNRT potentially aids in troubleshooting or in cases of loss of benefit.

The EF generated by a single electrode contact spreads in the intracochlear space thus stimulating not only the spiral ganglion cells in the proximity of the contact but also those that are further away. Stimulating-Current-Induced Non-Stimulating Electrode Voltage recordings are a generic term for measuring the voltage passively induced to non-stimulated electrodes, with reference to the ground electrode, to assess the EF along the array for a given stimulating contact (de Rijk et al. 2020). With Nucleus (Cochlear Ltd.) CIs, stimulating-current-induced non-stimulating electrode voltages can be measured both intra- and postoperatively with transimpedance matrix (TIM) measurements, and the voltages are normalized with the stimulating current resulting in ohm (Ω) units. Measuring TIM takes little time (2 to 3 min), and it is comfortable for the patient because the stimulation current can be set to low, often inaudible levels, whilst ensuring that the measured voltages are within the dynamic range of the detection electronics of the CI device (Leblans et al. 2022). Measuring TIM intraoperatively has the potential to help defining correct positioning of the CI electrode array within the cochlea, potentially reducing a need for intra- or postoperative radiological imaging (Vanpoucke et al. 2012; Hoppe et al. 2022; Söderqvist et al. 2023; Ayas et al. 2024).

Transimpedances measured on non-stimulating contacts can be used to determine the far-field component of access resistance by extrapolating the intracochlear EF toward the stimulating contact (Leblans et al. 2022). Berenstein et al. (2010) showed that the “effective” peak of the intracochlear EF related to psychophysical loudness was closer to the far-field than near-field access resistance. Assuming that this extrapolated far-field peak is related to activity elicited in the AN, Söderqvist et al. (2021) recently showed that the spread of intracochlear EF measured by TIM and the spread of neural excitation correlate in most patients. Moreover, the intraoperative effective peak amplitude of the EF thus assumed is inversely related to T-NRT (Söderqvist et al. 2022). Therefore, TIM potentially sheds light on how the AN reacts to the EF elicited by the electrode array on an individual level. Measuring TIM intraoperatively has been a clinical routine in our hospital since January 2019.

With both perimodiolar electrode arrays and arrays that are positioned closer to the lateral wall of the cochlea, T-NRTs decrease over the first few weeks postimplantation and stabilize within at least a few months showing little change after this (Lai et al. 2004; Björsne & Magnusson 2020). Without high-resolution postoperative imaging, comparisons of T-NRTs between individuals are somewhat difficult due to variation in distance between the electrode array and the spiral ganglion cells responding to electrical stimulation (Long et al. 2014) and the effect of individual cochlear morphology on the AN responsiveness (Söderqvist et al. 2022). There is a general increase in T-NRTs from the apical toward the basal electrode contacts (Cafarelli Dees et al. 2005; Spivak et al. 2011; Björsne & Magnusson 2020; Söderqvist et al. 2021).

It is customary to measure T-NRTs using different protocols for intraoperative and postoperative assessments. The intraoperative AutoNRT protocol uses a faster stimulation rate of 250 Hz to minimize measurement duration during CI surgery. A slower rate of 80 Hz is used postoperatively to reduce unpleasant sound sensations in awake recipients. The response of the AN to electrical stimulation decreases with increasing rate and becomes stochastic at around 2000 Hz (or pulses per second; He et al. 2017). Studies measuring T-NRT specifically with the two rates (80 and 250 Hz), however, have yielded somewhat varying effect sizes. Charasse et al. (2004) showed T-NRT to increase (i.e., the response of the AN to decrease) monotonically between the rates of 20 and 365 Hz, including the two rates used in the AutoNRT algorithm. Later longitudinal studies concluded that the effect of the two rates on T-NRT is statistically nonsignificant and markedly smaller than the decrease observed between intra- and postoperative assessments (Wesarg et al. 2010; Spivak et al 2011). The largest and statistically significant rate effect of about eight current levels (CL) was demonstrated in a recent study by Molisz et al. (2019).

The longitudinal decrease in T-NRT is generally larger than the corresponding decrease due to different stimulation rates between intra- and postoperative AutoNRT protocols, and there may be other factors that affect the responsiveness of the AN over time. It is unlikely that the onset of the electrical stimulation is related to the longitudinal decrease at large, because decreased T-NRTs may be recorded immediately at the initial CI activation when compared to the intraoperative values (Hughes et al. 2001; Spivak et al. 2011; Björsne & Magnusson 2020), leaving speculation about the role of fibrous tissue in this context. Measurable increases in access resistance already in the first weeks after the CI surgery have been documented, and their timescale is similar to the development of intracochlear fibrosis (Leblans et al. 2022). This led us to investigate if postoperative changes in the electrical circumstances within the cochlea were correlated with a change in the responsiveness of the AN.

The aim of this study was to assess whether changes in the responsiveness of the AN measured by T-NRT were related to changes in the peak and spread of the intracochlear EF measured by TIM during routine programming visits. A better understanding of their relationship could potentially help the clinician to predict changes in T-NRT profiles based on intraoperative assessment and a change observed between intra- and postoperative TIM measurements. In our experience, not all patients tolerate postoperative AutoNRT, while TIM can be more easily obtained.

## MATERIALS AND METHODS

This is a retrospective cohort study, which was approved by the institutional review board of the hospital.

### Study Subjects

One hundred forty-four ears of 113 consecutive patients (39 males, 74 females; mean age 32 years, range 0.7 to 90 years) were implanted with Cochlear Nucleus CI522 or CI622 implant with the Slim Straight electrode array (Cochlear Ltd.) between January 2019 and May 2022 in a tertiary referral center. Twenty-one patients were under 3 years of age. The etiology of the hearing loss is presented in Table 1. Forty-eight patients had bilateral CIs. Twenty-one patients were implanted simultaneously bilaterally. Twenty-seven patients were implanted sequentially, and 17 of those had received their first CI before the study period. Thus, 10 patients had both their sequentially implanted ears included in the study. In 109 patients, their severe-to-profound hearing loss was bilateral and in 4 patients it was unilateral. Intraoperative T-NRT and TIM recordings were available for 140 and 137 ears, respectively. Moreover, in three ears, the intraoperative TIM showed an unresolved open circuit. Postoperatively, T-NRTs were measured for 114 ears, for which there was a corresponding intraoperative T-NRT available in 108 ears. Thirty-four ears of 30 patients had both intra- and postoperative T-NRT and TIM data available.

**TABLE 1.** Etiology of the hearing loss in 113 patients receiving a cochlear implant

Etiology	n	%
Unknown	66	58.4
Genetic	19	16.8
Menière disease	10	8.8
Sudden sensorineural hearing loss	4	3.5
Meningitis	3	2.7
Otosclerosis	3	2.7
Perinatal asphyxia	3	2.7
Chronic otitis media	1	0.9
Congenital cytomegalovirus	1	0.9
Iatrogenic	1	0.9
Premature birth	1	0.9
Superficial siderosis	1	0.9
Total	113	

The surgical approach was round-window insertion in 134 ears and extended round-window insertion in 10 ears. In 130 ears, the CI electrode was fully inserted into the cochlea with the second (proximal) marker of the electrode array located at the level of the round window. In 14 ears, the insertion was partial between the distal and the proximal marker, but all electrode contacts of the active array were inserted into the cochlea. In 11 ears, the partial insertion was done with an intention to preserve residual hearing, while in 3 patients the insertion was left partial due to a sensation of increased resistance when advancing the electrode array into the cochlea.

A separate group of 10 ears of 8 consecutive patients (3 males, 5 females; mean age 30 years, range 0.9 to 63.7 years) implanted with the Slim Straight electrode array (CI622 implant) were included in the study to investigate the effect of stimulus rates of 250 and 80 Hz on intraoperative T-NRT. The surgical approach was round-window insertion in all ears. In nine ears, the electrode insertion was full, while in one ear, the insertion was partial due to a sensation of increased resistance.

### Impedance and eCAP Measurements

Intraoperative electrode impedances, TIM, and T-NRT, as well as the postoperative TIM, were measured by Custom Sound EP software with a Nucleus CP910 sound processor connected to a Nucleus programming pod (Cochlear Ltd.). For measuring intraoperative T-NRT, the AutoNRT protocol was used with a pulse width of 25 μsec, an inter-phase gap of 7 μsec, a stimulus rate of 250 Hz, and a starting value of 170 CL. Intraoperative measurements were made after electrode insertion, while the patient was still under general anesthesia. The TIM measurements were performed at a maximum level not exceeding the compliance limit or 230 CL. Intraoperative electrode impedances were measured at two time points for 123 ears of the total of 144 ears in the consecutive patient series: The first measurement was carried out before any other measurements, and the second after TIM and NRT measurements and their respective electrode conditioning. All four modes of electrode impedance (common ground, monopolar 1, monopolar 2 and monopolar 1 + 2) were measured.

The available postoperative electrode impedances, TIMs, and T-NRTs were collected from routine CI programming visits. Electrode impedances were available from all visits, however, the number of ears decreased gradually from the 123 ears intraoperatively to 75 ears for which approximately a 3-year electrode impedance measurement was available. For the 34 ears with both intra- and postoperative TIM and T-NRT data available, these measurements took place on average 101 (median 42; range 22 to 709) and 126 (median 62; range 23 to 427) days postimplantation, respectively.

Postoperative electrode impedances and T-NRTs with AutoNRT were measured by the Custom Sound fitting software with the Nucleus CP1000 or CP1150 sound processor connected to the Nucleus programming pod. In postoperative AutoNRT, pulse width was 25 μsec, inter-phase gap was 7 μsec, stimulus rate 80 Hz, and the starting CL was often inaudible and always well below the maximum comfortable level. For postoperative TIM with Custom Sound EP, the stimulus level was 109 CL to keep the possible sound sensation comfortable. TIM was measured both intra- and postoperatively with the default setting at the end of the first phase of the biphasic stimulation pulse.

The intraoperative T-NRTs were adjusted for the rate difference in the AutoNRT protocols based on the available average absolute values of T-NRTs measured at 250 and 80 Hz (Spivak et al. 2011; n = 21; Molisz et al. 2019, n = 35) or their ratio (Charasse et al. 2004, n = 6) in the literature, and based on a separate group of 10 ears measured intraoperatively with the two rates at our hospital during the present study. In our group of 10 ears, the measurement order was counterbalanced so that for half of the ears, T-NRT was measured first with the 250 Hz rate for all electrode contacts, followed by a measurement with the 80 Hz rate on nine contacts along the electrode array. The order was reversed for the other half. Electrode conditioning was utilized in all measurements. A mean electrode-independent adjustment factor was computed as a ratio between the T-NRT measured with slower (80 Hz) and faster (250 Hz) stimulation rate, weighted by the number of ears in each study. The ratios computed from Spivak et al. (2011) and Molisz et al. (2019) were 0.982 and 0.957, respectively, and 0.981 as estimated from Figure 7 of Charasse et al. (2004).

Because the individual postoperative programming visits took place at different time intervals from CI surgery and the first impedance measurement, the electrode impedances were interpolated over time for visualization purposes. In the interpolation, the resolution was 14 min for the intraoperative data, 1 day up to 50 postoperative days, 10 days between 50 and 100 postoperative days, and 200 days after 100 postoperative days. This allowed for illustration of the evolution of mean electrode impedances over time without averaging out details due to individual differences in postoperative appointment times. For AutoNRT and TIM, the closest postoperative measurements in time were selected for comparison with their intraoperative counterparts.

The lower stimulus level in the postoperative TIM measurement resulted in smaller voltages in the non-stimulating contacts compared to the intraoperative measurement rendering comparisons of far-field access resistance in absolute units impossible. To allow for a comparison of the intracochlear EF characteristics between intra- and postoperative assessment, we parameterized each TIM measurement based on computations described by Berenstein et al. (2010). The decay of the intracochlear EF for a given stimulating contact was first modeled with exponential functions. Transimpedance $Z_{far}$, which is related to electric potential along the array for a given stimulating current, can be estimated from:

$$\begin{array}{l} Z_{far}=A\cdot e^{-\frac{x}{\lambda }}+dc, \end{array}$$ (1)

where A is amplitude, x is distance of a recording contact from the stimulating contact in millimeters (mm), *λ* is a length constant (in mm) separately for the apical and basal directions, and dc is a direct current component or an offset from zero. The sum of squares of the error between the measured and modeled EF on the non-stimulating contacts was minimized by optimizing the parameters A, $\lambda_{apical}$, $\lambda_{basal}$ and dc with the *fminsearch* function of MATLAB, version 24.2.0 (MathWorks Inc., Natick, MA, USA). The parameterization yielded (1) far-field impedance at the location of the stimulating electrode ($Z_{far}=A+dc$, when x=0), (2) a dc plateau representing a far-field residual that the EF approaches asymptotically far away from the stimulating electrode, and (3) length constants ($\lambda_{apical}$ and $\lambda_{basal}$) that describe the decay of the EF to both apical and basal directions from the stimulating contact. We further assumed that the effective transimpedance relevant to the responsiveness of the AN is equal to *Z*_far_, when x=0.

**Fig. 1.**
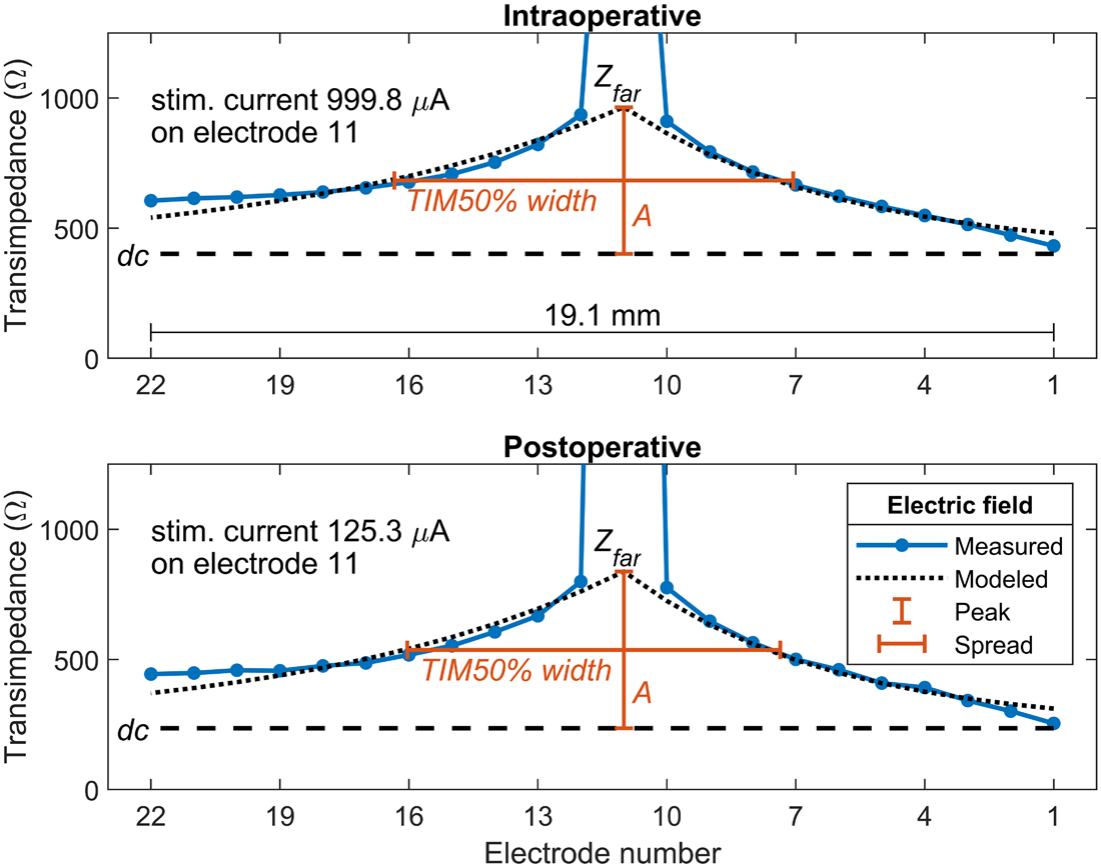
Examples of intra- and postoperative EF profiles on electrode 11 with a modeled effective peak amplitude (A) and a dc-value representing a stimulus-current dependent plateau for each profile. The peak and the spread of the intracochlear EF are represented by A and TIM50%, respectively (marked in orange). EF indicates electric field.

On the basis of this, we then took amplitude A (in Ω) from Eq. (1) to representing a far-field peak of the EF at the stimulating contact compared to its plateau ($A=Z_{far}-dc$, when x=0), independent of stimulation CL. Moreover, we computed TIM50% width to represent the spread of the effective transimpedance, that is, the width of the transimpedance (in mm) where the *Z*_far_ is 50% of its peak amplitude relative to the dc offset, in the same manner as Söderqvist et al. (2021, 2022). With amplitude A and TIM50% width, we were able to examine if the changes in T-NRT were related to the changes in the characteristics of the intracochlear EF. Figure 1 presents an example of the measured and the modeled EF profile characteristics on a single electrode contact for intra- and postoperative assessment, as well as their corresponding EF peak and spread parameters.

### Statistical Analyses

A linear mixed-effects (LME) model with electrode impedance as a response variable, stimulation mode (monopolar 1, monopolar 2, and common ground), and measurement time point (postoperative days) as independent fixed-effect variables, and ear as a random-effect variable was applied to inspect the evolution of electrode impedance over postoperative days. For the monopolar 1 + 2 data representing the most often used stimulation mode in Nucleus CIs, the same analysis was performed by replacing stimulation mode with electrode section (apical, middle, and basal; electrodes 16 to 22, 8 to 15, and 1 to 7, respectively) as a fixed-effect variable. Impedances from the intraoperative and first postoperative sessions were excluded from these analyses.

A paired samples *t* test was used to compare intra- and postoperative T-NRTs. The evolution of postoperative T-NRTs was analyzed with three LME models separately for the apical, middle, and basal sections with T-NRT as a response variable, electrode contact and measurement time point as fixed-effect variables, and ear as a random-effect variable. Because the majority of the postoperative T-NRTs were measured within the first 100 days postoperatively, measurement time point was log-transformed for these LME models. A two-way repeated-measures analysis of variance (ANOVA) with stimulation rate and electrode contact as dependent variables and T-NRT as an independent variable was used to analyze the effect of the AutoNRT protocol on intraoperative T-NRTs for the separate group of 10 ears.

Last, a multivariate ANOVA with T-NRT, EF peak (amplitude A) and EF spread (TIM50% width) as dependent variables, and electrode section (apical, middle, basal) and measurement time point (intra- and postoperative) as independent variables was applied to inspect changes in T-NRT and the related changes in the intracochlear EF in different parts of the electrode array over the two available time points. Multiple comparisons were adjusted with the Bonferroni correction. The Spearman correlation coefficient was computed for postoperative T-NRT and measurement time point, and for a change in T-NRT and the corresponding change in EF peak and EF spread between intra- and postoperative measurements. An alpha level of 0.05 was considered for statistical significance. The statistical tests were performed with IBM SPSS Statistics for Windows, Version 29 (IBM Corp., Armonk, NY, USA), and the LMEs and correlation coefficients were computed with MATLAB, Version 24.2.0 (R2024b; MathWorks Inc., Natick, MA, USA).

## RESULTS

The aim of the study was to assess whether changes in the responsiveness of the AN measured by T-NRT were related to changes in the peak and spread of the intracochlear EF measured by TIM during routine programming visits. We also analyzed the evolution of interpolated electrode impedances for different stimulation modes and in different sections of the electrode array. In addition, we measured T-NRT for a smaller group of subjects with the two AutoNRT protocols, and together with earlier published data, attempted to factor in the effect of stimulation rate on the observed change in T-NRT between intra- and postoperative assessments.

**Fig. 2.**
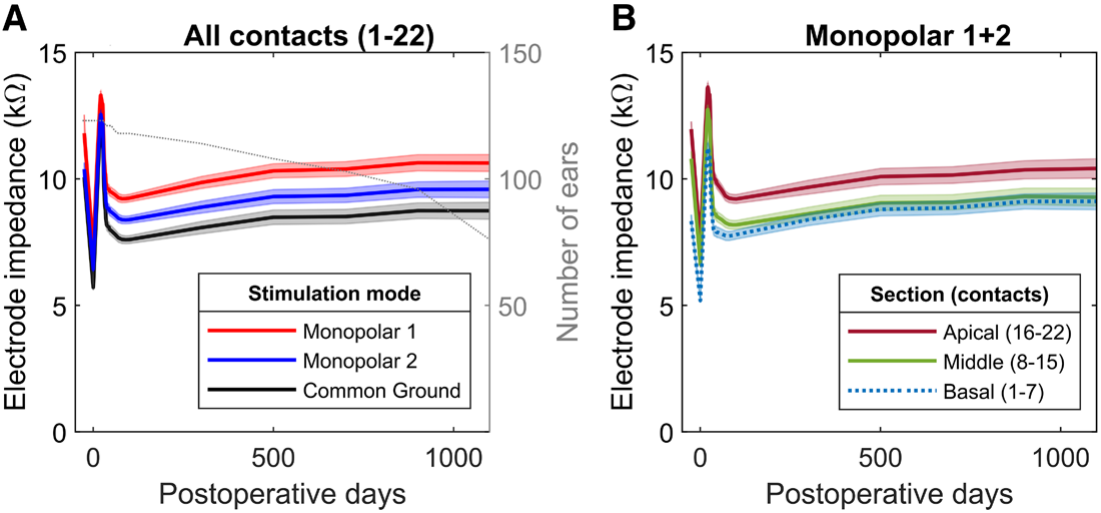
A, Mean ± SD interpolated electrode impedances over time by three different measurement modes for all 22 electrode contacts across the Slim Straight electrode array. The gray line represents the number of measured ears at each time point. The SD of the impedances increased over time probably because the number of measured ears per postoperative time point (gray dotted line) decreased from 123 to 75 during the follow-up. B, Mean ± SD interpolated electrode impedances over time measured by monopolar 1 + 2 (equivalent to monopolar 2) mode for all 22 electrode contacts divided in three groups depending on their location along the array. In both diagrams, the furthest point on the left marks the initial intraoperative electrode impedances before conditioning, followed by a trough during the second intraoperative measurement, which was after measuring TIM and AutoNRT. For illustrative purposes, the period including the two intraoperative measurements is spread out over time. The following peak represents the electrode impedances measured at the beginning of the CI activation visit before device switch-on. After the activation of the CI, the impedances increased gradually over time. Ears with two intraoperative impedance measurements were included in the diagrams. CI indicates cochlear implant; TIM, transimpedance matrix.

Figure 2 illustrates the change in the interpolated electrode impedances as a function of postoperative days, averaged over ears (mean ± SD). There was an instantaneous drop in electrode impedance between the two intraoperative measurements, that is, before and after TIM and NRT measurements. The following peak is aligned with device activation, followed by an immediate drop due to the onset of electrical stimulation and then by a gradual rise over time. The decreasing number of ears per postoperative time point may explain the increasing variance in electrode impedance at later postoperative measurements. Figure 2A illustrates the change in impedance averaged over all electrode contacts for monopolar 1, monopolar 2, and common ground stimulation modes. In the LME model, there was a statistically significant effect of stimulation mode and time point both on the intercept (*p* < 0.001) and the slope (*p* < 0.001 and *p* = 0.001). The interaction of the fixed effects was, however, nonsignificant, suggesting that there were no stimulation-mode dependencies in the evolution of electrode impedance over time. Adding the interaction term to the model did not improve prediction accuracy based on the theoretical likelihood ratio test. Figure 2B illustrates the change in impedance in different sections of the electrode array. In the LME model, there was again a statistically significant effect of both electrode section (*p* < 0.001) and time point (*p* < 0.001), but in contrast to Figure 2A, their interaction was also significant (*p* < 0.001). This suggests that the increase in impedance depends on electrode section, which can be seen as a somewhat greater increase over time for the basal section of the array in the interpolated data of Figure 2B. The random variable (ear) had a significant effect both on the intercept and the slope of both LME models, suggesting that there are individual characteristics in overall electrode impedances and how they evolve over time.

**Fig. 3.**
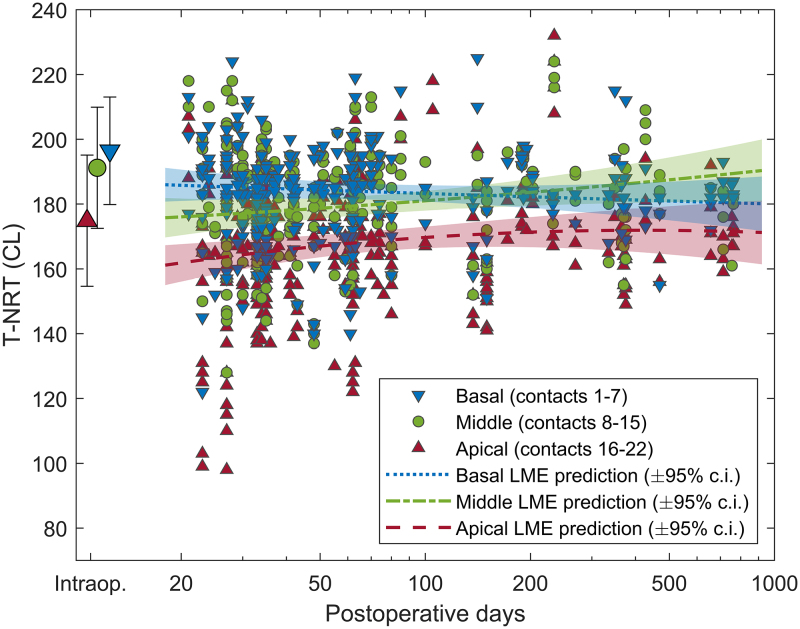
Intraoperative T-NRTs (mean ± SD) and the corresponding postoperative T-NRTs in different sections of the electrode array that were available for 108 ears out of the total of 144 consecutively implanted ears. Intraoperatively, on average 21.23 (range 4 to 22) contacts per ear were measured. The data for apical, middle, and basal sections of the electrode array are specified with different colors and symbols. Linear mixed-effects model predictions with their 95% confidence intervals are presented for the three sections. NRT indicates neural response telemetry.

The intraoperative T-NRTs (mean ± SD) and the corresponding postoperative T-NRTs in different sections of the electrode array for 108 ears that had both intra- and postoperatively at least one T-NRT available are illustrated in Figure 3. There was a statistically significant decrease of 10.85 ± 13.85 CL (mean ± SD) between the mean intra- and postoperative T-NRT (*p* < 0.001, paired samples *t* test) obtained with the stimulation rates of 250 and 80 Hz, respectively. The LME models applied to the postoperative T-NRTs separately for the apical, middle and basal sections of the electrode array revealed that there were statistically significant effects for electrode contact, measurement time point and their interaction on the intercept (*p* < 0.001) and the slope (*p* = 0.003, *p* = 0.03, and *p* = 0.008, respectively) for the apical section of the array. These effects were nonsignificant for the middle and basal sections, suggesting that after an initial decrease, T-NRT remains stable in these parts of the electrode array. LME model predictions with their 95% confidence intervals are plotted in Figure 3 separately for the three sections. The effect of the random variable (ear) was significant in all three LME models.

The mean intraoperative T-NRT (±SD) for the separate group of 10 ears with nine contacts along the electrode array, at stimulation rates of 250 and 80 Hz, is listed in Table 2. In the repeated-measures ANOVA, the main effects of stimulation rate and electrode contact were significant (*p* < 0.001 for both), and their interaction was nonsignificant. The mean difference (±SD) between the rates was 2.29 ± 5.74 CL, corresponding to an average ratio of 0.988 between T-NRT measured with 80 and 250 Hz stimulation rates. Because the interaction term was nonsignificant in this group of patients, in Spivak et al. (2011) and in Molisz et al. (2019), the intraoperative T-NRTs were multiplied with the adjustment factor, independent of electrode contact, to yield more comparable values with the postoperative T-NRTs in our clinical data. The final adjustment factor weighted by the number of ears across studies was 0.970. After multiplying each intraoperative T-NRT with this factor, the decrease in postoperative T-NRT was 5.317 ± 13.55 CL (mean ± SD), which was still statistically significant (*p* < 0.001, paired samples *t* test).

**TABLE 2.** Mean ± SD intraoperative T-NRT in CL recorded for nine electrode contacts in 10 ears receiving the Slim Straight electrode array with 250 and 80 Hz stimulus rates, as well as their ratio

	22	19	16	13	11	8	6	3	1
250 Hz	174.1 ± 23.3	176.8 ± 15.3	174.1 ± 19.0	187.8 ± 22.2	194.5 ± 16.0	203.2 ± 12.9	201.0 ± 28.0	198.1 ± 17.2	197.3 ± 20.4
80 Hz	170.9 ± 24.8	172.9 ± 16.1	173.4 ± 18.7	182.2 ± 21.1	192.2 ± 16.7	202.1 ± 15.7	200.8 ± 18.8	194.6 ± 18.1	197.2 ± 18.6
Ratio (80/250)	0.982	0.978	0.996	0.970	0.988	0.995	0.999	0.982	0.999

CL, current level; NRT, neural response telemetry.

**Fig. 4.**
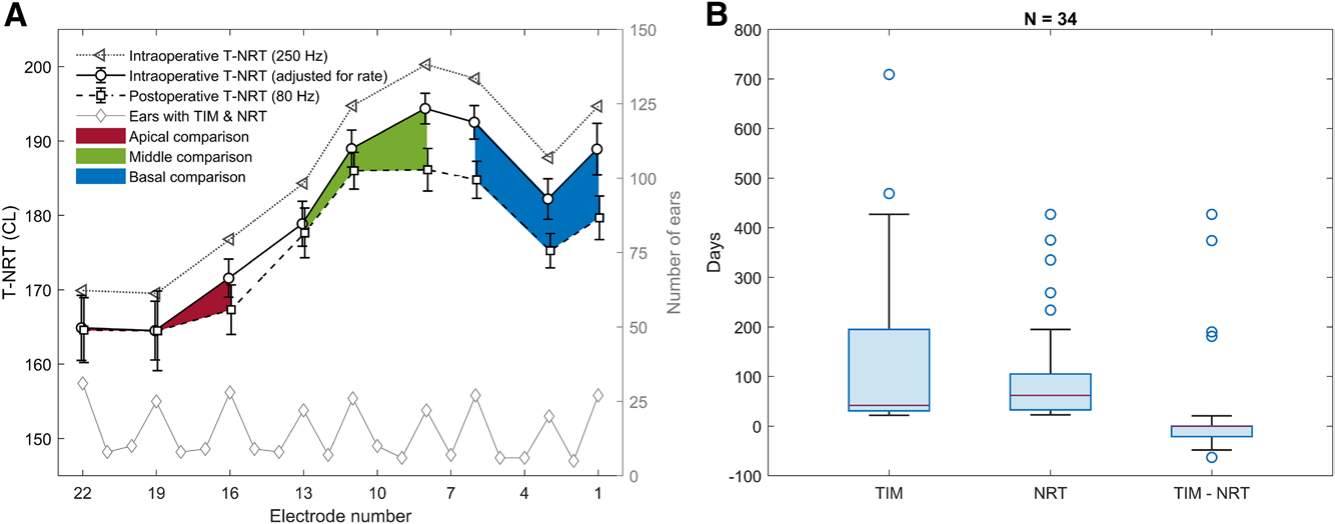
Panel A shows intra- and postoperative T-NRT (mean ± SEM) with their original rates and the intraoperative T-NRT adjusted for the rate difference for electrode contacts where a minimum of 20 ears were measured intra- and postoperatively. The total number of ears where both an intra- and postoperative T-NRT was available for a given contact is plotted at the bottom (n = 31, 25, 28, 22, 26, 22, 27, 20, and 27 for electrodes 22, 19, 16, 13, 11, 8, 6, 3, and 1, respectively). Panel B shows the distribution of the time point for postoperative TIM and NRT measurements in days, as well as their difference. NRT indicates neural response telemetry; SEM, standard error of mean; TIM, transimpedance matrix.

Figure 4A shows the mean ± standard error of mean of intra- and postoperative T-NRTs measured with their original rates, the intraoperative T-NRT adjusted for the rate difference, as well as the number of ears on the right *y*-axis for which an intra- and postoperative TIM and T-NRT for a given electrode contact was available. Electrode contacts with at least 20 (and up to 31) ears are included in the mean values of different sections in the array. The total number of ears in the mean values of Figure 4A is 34. T-NRT as well as the difference between the intra- and postoperative T-NRT appears to increase from apical to basal direction, also when individual intraoperative T-NRTs are adjusted for stimulation rate. For 15 out of the 34 ears, postoperative TIM and NRT were available from the same appointment, for 11 ears TIM was measured at an earlier appointment and for 9 ears at a later appointment than NRT. Boxplots in Figure 4B illustrate the distribution of postoperative TIM and NRT measurement days from CI surgery, and the right-most boxplot is their difference. The median difference was 0 days, and the interquartile range from −21 to 0 days.

**Fig. 5.**
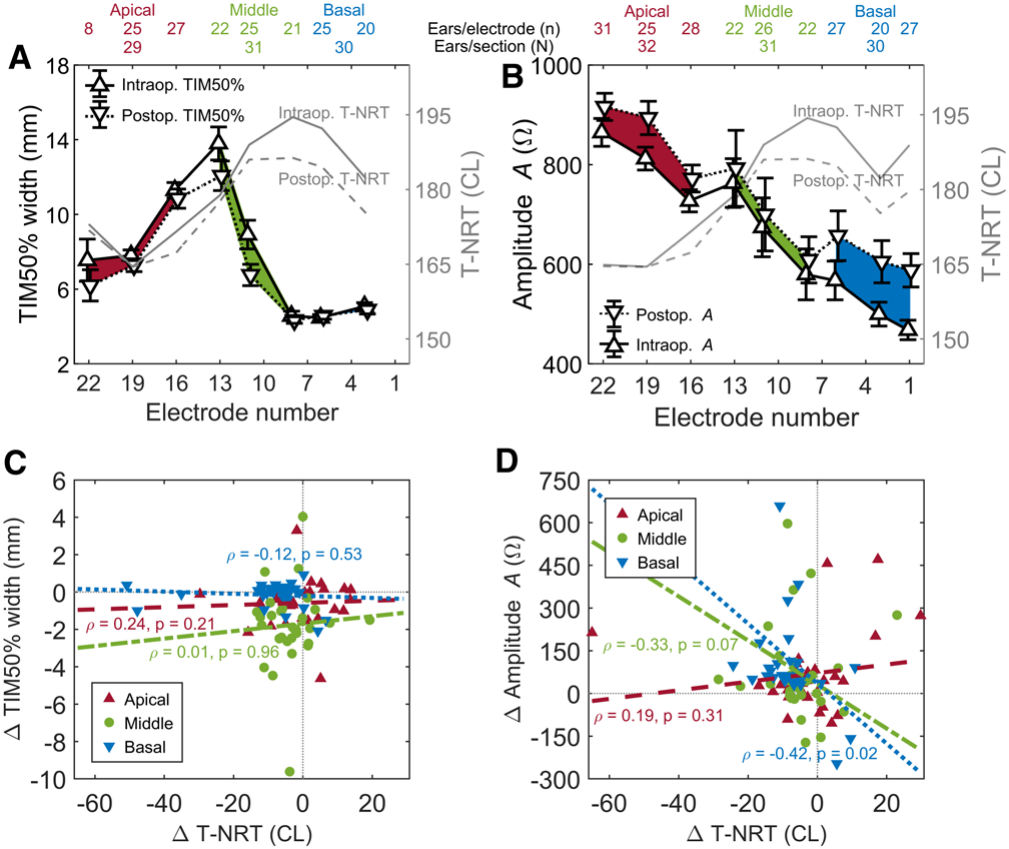
Panel A shows the mean ± SEM intracochlear EF width (i.e., TIM50% width, see text for details) intra- and postoperatively with the corresponding T-NRTs. The red section corresponds to apical, green to middle and blue to basal comparison. Panel B shows the mean ± SEM intracochlear EF peak (A, see text for details) intra- and postoperatively with the corresponding T-NRTs with a similar color coding to panel A. In both panels A and B, the data are plotted only for those ears and electrode contacts with intra- and postoperative T-NRT (solid and dashed gray lines, respectively) and a corresponding computational EF parameter was available. The number of measured ears per electrode contact and array section is shown above the panels A and B. The difference averaged across electrode section (apical, basal, and middle) in T-NRT, EF width, and EF peak for each ear with the corresponding correlation coefficients and linear regression lines for visualization are shown in panels C and D, respectively. EF indicates electric field; NRT, neural response telemetry; SEM, standard error of mean.

In Figure 5, the difference between intraoperative and postoperative assessment is explored further, not only for rate adjusted T-NRT but also for EF spread (i.e., TIM50% width, see Fig. 5A) and EF peak (i.e., amplitude A, see Fig. 5B). Computing EF spread for electrode contact 1 was unfeasible because its peak was too low and transimpedance did not reach 50% of its peak amplitude. To provide independent samples for statistical analyses, T-NRT, TIM50% width and amplitude A values were averaged within the three electrode sections for each ear. In the averaged data, there were two outlying EF peak values with a *z*-score higher than 3.29 in the middle section of the array, and these outliers were removed from the subsequent statistical analyses of the data presented in Figures 5A,B. The mean change in T-NRT, EF spread and EF peak was −4.9 CL, −0.84 mm and 83 Ω, respectively, between intra- and postoperative measurements, suggesting an increased responsiveness of the AN, and a narrowing width and increased peakedness of the intracochlear EF. In the multivariate ANOVA, the main effect of measurement time point was statistically significant for T-NRT (*p* = 0.035), EF peak (*p* < 0.001), and EF spread (*p* = 0.006). Bonferroni-adjusted pairwise comparisons revealed that T-NRT decreased significantly between intraoperative and postoperative measurement in the basal (*p* = 0.043) but not in the apical (*p* = 0.822) and middle section (*p* = 0.157) of the electrode array. The intracochlear EF spread narrowed significantly between intra- and postoperative measures in the middle section of the electrode array (*p* < 0.001) and the EF peak increased significantly in the basal section (*p* = 0.003).

Last, the difference in the mean value within each electrode section between intra- and postoperative T-NRT, EF spread, and EF peak was computed for each ear. These differences (postoperative minus intraoperative value) are plotted as a change in T-NRT versus the corresponding change in EF spread in Figure 5C, and as a change in T-NRT versus the corresponding change in EF peak in Figure 5D for contacts in the apical, middle, and basal sections of the electrode array. The dotted gray lines at a value of zero denote no difference. Most of the ΔT-NRT and ΔTIM50% width values are below zero, while for ΔA they are above zero, indicating a postoperative increase in the responsiveness of the AN (i.e., a lower T-NRT), narrowing of the EF spread, and increased peakedness of the EF at and around the stimulating contact. The corresponding Spearman correlation coefficients and their *ρ*-values are added alongside the data. The linear regression lines are plotted for visualization. The change in EF spread was not correlated with a change in T-NRT (*p* ≥ 0.21). There was, however, a statistically significant negative correlation between the change in EF peak and the change in T-NRT in the basal section (*ρ* = −0.42, *p* = 0.02) of the electrode array.

## DISCUSSION

The main finding of the present study is a significant negative correlation between the change in EF peak and the change in eCAP thresholds between intra- and postoperative assessments in the basal section of the electrode array. This suggests that the increased postoperative peakedness of the intracochlear EF in the basal section of the array is correlated with an increased responsiveness of the AN.

Clinical electrode impedances were inspected for longitudinal changes, and we found no stimulation-mode dependencies in their evolution over time. This suggests that changes in electrode impedances are mostly of intracochlear origin, where the basal section of the electrode array exhibited the largest increase in impedance. The overall evolution and the more pronounced increase in the basal section agree with earlier studies (Hughes et al. 2001; Wimmer et al. 2022). Indeed, histopathological studies have shown that new tissue growth after cochlear implantation is greater at the basal turn of the cochlea (Li et al. 2007; Seyyedi & Nadol 2014). When inspecting the time course of electrode impedance in more detail, we noticed an immediate decrease between the intraoperative electrode impedances, that is, before and after the electrode array was electrically stimulated for the first time. This decrease is most likely due to a decrease in polarization impedance. At the CI activation visit, a new peak in electrode impedance was noted, and it seems to be at a higher level than the initial intraoperative impedance. This may be explained by another rise in polarization impedance due to a non-stimulating electrode array, and an additional increase in access resistance caused by fibrous tissue formation. After the onset of CI stimulation at the activation visit, the electrode impedances show again a distinct decrease in agreement with decreasing polarization impedance, which is followed by a gradual increase possibly due to slowly increasing fibrous tissue formation around the electrode array. The number of ears with available electrode impedances at later postoperative visits decreased in our study, and some of the increase may be due to individual differences in the interpolated impedance values of Figures 2A,B. Nevertheless, the trend is similar to earlier studies on stimulation-related decreases in polarization impedance and fibrosis-related increases in access resistance (Tykocinski et al. 2005; Newbold et al. 2010, 2014; Leblans et al. 2022; Wimmer et al. 2022). The two intraoperative electrode impedance measurements (before and after electrical stimulation with the CI) of the present study shed some additional light on the role of polarization impedance in clinical electrode impedances.

Our finding that eCAP thresholds decrease between intra- and postoperative assessment and that they show no significant change in the middle and basal sections over the postoperative period agrees with earlier studies where it was found that the eCAPs stabilize at least within a few months (Lai et al. 2004; Molisz et al. 2015; Björsne & Magnusson 2020). However, eCAP thresholds increased over time in the apical section following device activation in our data (see Fig. 3), which agrees with evidence for delayed stabilization for apical contacts (Molisz et al. 2015) and larger absolute differences reported between T-NRTs measured intraoperatively and at initial activation compared to later time points (Björsne & Magnusson 2020).

It has been suggested that fibrous tissue forming over time around the electrode affects the electrical transmission from the electrode to the AN (Hughes et al. 2001). More specifically, the differences in the fibrous tissue encapsulation around the electrode array within the cochlea have been proposed to explain the postoperatively higher impedances at the basal part of the cochlea compared to the apical end (Saunders et al. 2002). The development of more atraumatic implantation techniques and thinner electrode arrays today may lead to less intracochlear fibrous growth and new bone formation compared to the previous decades (Ishai et al. 2017). The damage to the lateral wall of the cochlea has been found to correlate with the extent of fibrosis formation and new bone growth for lateral-wall electrodes (Li et al. 2007; Kamakura & Nadol 2016; Geerardyn et al. 2024). However, insertion trauma alone seems not to be a sole determinant of the formation of fibrous tissue (Kamakura & Nadol 2016). In the present study, we found that the postoperative eCAP thresholds decreased significantly from the intraoperative values in the basal section of the cochlea.

There is a small but statistically significant increase in the transimpedance across all electrode contacts between intraoperative and 3-month follow-up visit, and for a subset of electrode contacts in the apical and middle sections of the array between intraoperative and CI activation probably due to fibrosis and inflammation (Hoppe et al. 2022). In our data, the postoperative intracochlear EF peak increased significantly from the intraoperative value in the basal section and the EF spread narrowed in the middle section. Because the cross-sectional diameter of the scala tympani is smaller in the apical part of the cochlea compared to the basal part, fibrous tissue growth most likely affects the intracochlear EF, and the estimates of its peak and spread in the present study, differ in different sections of the cochlea. In the basal part, where fibrous growth is strongest, fibrous tissue has been suggested to inhibit the longitudinal flow of current along the electrode array, resulting in increases in far-field resistance (Leblans et al. 2022). Increased far-field resistance over time in contacts adjacent to the stimulating electrode contact would lead to an increased EF peak in our data. In the middle section, further away from the round window, potential fibrous tissue formation appears to mostly decrease the EF spread, and perhaps provide more focused stimulation increasing its effectiveness, although this effect was not statistically significant. Because we found a negative correlation between the change in EF peak and the change in eCAP thresholds, this may explain that the postoperative eCAP threshold decreases more in the basal part of the cochlea from its intraoperative value.

On the basis of our findings, we suggest that fibrous tissue leads to a narrowing width and increased peakedness of intracochlear EF which in turn leads to lower eCAP thresholds at least in the basal section of the electrode array. However, it is yet to be resolved how fibrous tissue enclosing the electrode array affects the response in NRT measurements. It may be assumed that the effect of fibrous tissue is not identical for the EF produced by the stimulating contact, which is recorded on non-stimulated contacts along the array, and for EF of the response, that is, eCAPs derived from the AN, which are recorded on a contact that is typically two contacts away to the apical direction. There is recent evidence that electrode impedances are correlated with subjective threshold and maximum comfort levels, and that their predictive value in CI programming is greater than that of T-NRT (Zarowski et al. 2020, 2023). There are, however, large differences between clinics and clinicians in setting the CI stimulation levels based on subjective loudness perception of the recipient (Vaerenberg et al. 2014), whereas AutoNRT presumably will produce more uniform outcomes, decreasing the variability due to the clinician. The relatively low, but statistically significant correlation between changes in the EF peak and T-NRT in the basal part of the cochlea may therefore help in understanding the role of the intracochlear EF in the responsiveness of the AN.

Our study evaluated retrospective clinical data, where different protocols were used for both TIM and AutoNRT measurements intra- and postoperatively. The EF analyses of our study were based on computational parameters of the EF peak and spread that were measured with considerably larger stimulating CLs intraoperatively compared to the postoperative measurements, resulting in units relative to the dc plateau. Therefore, the amplitude parameter A is unlikely to be the only relevant parameter in estimating the responsiveness of the AN, despite its statistically significant negative correlation with T-NRT. The optimization of these parameters relies on a monotonic, exponential decay of the intracochlear EF, which may not be the case in all individuals (e.g., in the case of electrode malposition; Ayas et al. 2024). In our clinical practice, TIM measurements are systematically reviewed, and postoperative imaging is performed only on recipients with abnormal results. No such abnormalities were present in the recipients of this study. In AutoNRT, the effect size of the two different stimulation rates on T-NRT varies in the literature (Wesarg et al. 2010; Spivak et al. 2011; Molisz et al. 2019), but it seems to account only part for the difference between intra- and postoperative T-NRT. While multiplying T-NRTs with an adjustment factor affects lower and higher values (e.g., in the apical and basal sections) differently in terms of CL, there is individual variability in T-NRT with different rates (Charasse et al. 2004; Spivak et al. 2011; Molisz et al. 2019; present study) that cannot be accounted for by a generic factor. Nevertheless, there is no evidence for significant interaction of rate with electrode contact, which suggest that the use of a single adjustment factor along the electrode array is reasonable. These uncertainties are a potential reason for generally smaller correlation coefficients than what has been published for subjective stimulation levels and absolute impedance values (Zarowski et al. 2020, 2023).

The timing for the postoperative measurements varied considerably in our patients, even though the closest measurements in time were selected for analyses. T-NRT seems to stabilize sooner in the basal section compared to the apical section (see Fig. 3 and Molisz et al. 2015), and this may explain stronger correlations in this part of the electrode array, as there are sizable changes, especially in the near-field access resistance early on after CI activation (Leblans et al. 2022). The low rate of postoperative TIM measurements is another weakness of this study. This is likely due to an older sound processor and different software (Custom Sound EP) required at the fitting appointments. On the other hand, one of the strengths of this study was that each of the measured ears served as its own controls assuming that the location of the intracochlear electrode array and other individual characteristics remained unchanged between the intra- and postoperative measurements. Further research with TIM measurements at several parts of the biphasic pulse and at fixed intervals after CI activation would potentially help with differentiating polarization impedance and the near-field component of access resistance from its far-field component, revealing a more detailed understanding of their relationship with responsiveness of the AN.

## CONCLUSIONS

Electrode impedances exhibited a long-term increase aligned with the onset of electrical stimulation and the time course of intracochlear fibrous tissue formation after CI surgery. The long-term increase in electrode impedance was more pronounced in the basal section of the electrode array. A novel finding of this study was the negative correlation between the change in EF peak and the change in T-NRT in the basal sections of the electrode array, that is, the increased postoperative peakedness of the intracochlear EF correlated with the increased responsiveness of the AN. The more peaked intracochlear EF may be due to fibrous tissue formation around the electrode array.

## ACKNOWLEDGMENTS

This study was supported by the HUS Helsinki University Hospital Research Grant (TYH2023335), HUS Helsinki University Hospital departmental research grants, and Tauno Palva Foundation.
